# Effect of antimicrobials administered via liquid feed on the occurrence of sulphonamide and trimethoprim resistant Enterobacteriaceae: case-control study

**DOI:** 10.1186/s40813-017-0067-0

**Published:** 2017-10-03

**Authors:** Oliver Heller, Roger Stephan, Sophie Thanner, Michael Hässig, Giuseppe Bee, Andreas Gutzwiller, Xaver Sidler

**Affiliations:** 1 Department for Farm Animals, Division of Swine Medicine, University of Zurich, Winterthurerstrasse 260, 8057 Zurich, Switzerland; 2 Institute for Food Safety and Hygiene, University of Zurich, Winterthurerstrasse 272, 8057 Zurich, Switzerland; 3 Department for Farm Animals, Section for Ambulatory Service and Herd Health, University of Zurich, Winterthurerstrasse 260, 8057 Zurich, Switzerland; 4 Institute for Livestock Sciences, Agroscope, Tioleyre 4, 1725 Posieux, Switzerland

**Keywords:** Antimicrobial resistance, Oral group therapy, Sulphonamide, Trimethoprim, Enterobacteriaceae, Liquid feeding, Fattening pigs

## Abstract

**Background:**

Drugs for the treatment of groups of pigs receiving liquid feed are frequently mixed into the feed and administered via the pipelines of the feeding installations. In-feed antimicrobials may select antimicrobial resistant strains among the bacteria which form the biofilm of these pipelines and are shed into the liquid feed.

**Objective and methods:**

In order to evaluate the risk of selecting antimicrobial resistant bacteria in the biofilm of liquid feeding installations, the effect of the administration of antimicrobials via the pipelines on the occurrence of antimicrobial resistance in the feed was examined in a case-control study. A premix containing either sulphonamide plus trimethoprim or sulphonamide plus chlortetracycline plus tylosin or chlortetracycline was administered via the pipelines to each batch of bought-in fattening pigs in 7, 3 and 3 case farms respectively, whereas antimicrobials had not been administered via the liquid feeding installation for at least 2 years in the 14 control farms. Enterobacteriaceae and sulphonamide-trimethoprim resistant Enterobacteriaceae were counted in twelve and eight feed samples collected in each case and in each control farm respectively during one fattening period. The semiparametric Generalized Estimating Equations (GEE) method was used for the statistical data analysis.

**Results:**

The ratio of sulphonamide and trimethoprim resistant to total Enterobacteriaceae was higher in the feed of the case farms compared to the control farms (*P* < 0.001) and did not decrease after treatment during the fattening period.

**Conclusion:**

The administration of antimicrobials via the liquid feeding installation selects antibiotic resistant bacteria in the biofilm lining the pipelines, which may contaminate the liquid feed for extended periods and transmit their resistance genes to the gastrointestinal flora of the pigs. Alternatives to the administration of antimicrobials via pipelines of liquid feeding installations for group treatment should be developed.

## Background

Because a high antimicrobial use is associated with high levels of antimicrobial resistance (AMR) [1], the prudent and reduced use of antimicrobials in farm animals has become an important goal. Between 2008 and 2014, the annual amount of antimicrobials used for disease prevention and treatment in Swiss farm animals decreased by almost a third, from 71 to 48 tons [2]. In 2014, antimicrobial premixes for in-feed use accounted for 60% of the total amount of antimicrobials used in farm animals. Among pigs, newly weaned pigs and pigs entering a fattening unit are the two groups that are most frequently treated with antimicrobials [3]. Although the routine prophylactic use of antimicrobials is strongly discouraged [4], oral group treatment for disease prevention is still the main indication (79%) for antimicrobial use in fattening pigs in Switzerland [5], followed by oral group therapy in disease outbreaks (18%) and individual treatment of sick animals (3%). In Switzerland, the most commonly used antimicrobial drug for oral group treatment contains sulphathiazole, sulphadimidine and trimethoprim, being followed by a combination containing chlortetracycline, sulphadimidine and tylosin, and drugs containing either chlortetracycline or colistin.

“In Switzerland, by-products of the food industry, in particular whey, a by-product of the cheese production, are part of most pig fattening rations. The most economical way to use these by-products is to mix them on the farm with commercial complementary feeds and administer the mixed feeds via liquid feeding installations.” Dry feed and liquid (usually whey or water) are mixed in the mixing tank and pumped through a ring line to the drop pipes and into the feed troughs. In these liquid feeding installations, the liquid feed inside the ring line remains there between two feeding times, being diluted with water in some farms, and is pumped back into the mixing tank during the next mixing process. The lines of liquid feeding systems are coated with a biofilm, consisting of a community of microorganisms which stick together and produce a slime composed of extracellular polymeric substances. The administration of antimicrobials via liquid feeding installations poses a risk of selecting antimicrobial resistant bacteria in the biofilm. The latter may therefore be regarded as a potential reservoir of resistant bacteria. At any time, parts of the biofilm can be detached by mechanical forces or various biological processes [6] and disperse in the liquid feed. Antibiotic resistant bacteria originating from the biofilm are therefore ingested by pigs, thus adding further AMR genes to the AMR gene pool already present in the pig gut. According to the WHO [7], major gaps exist in the surveillance related to the emergence of AMR in foodborne bacteria. The effect of antimicrobials administered via liquid feeding installations on the AMR prevalence in the liquid feed has to our knowledge not been studied yet. The aim of this case-control-study was to assess the effect of the administration of three different antimicrobial drug formulations via liquid feeding installations on the prevalence of sulphonamide + trimethoprim resistant Enterobacteriaceae used as indicator bacteria in the liquid feed for pigs.

## Methods

### Study design, farm management investigation and sample collection

A total of 27 pig fattening farms located in different regions of Switzerland which used computer-assisted liquid feeding installations were included in the case-control study. In the 13 case farms, antimicrobials had been administered in-feed via the feed pipeline in every fattening period lasting about 3 months for at least the last 2 years prior to the study. In the 14 control farms, antimicrobials had not been administered via the pipeline for at least 2 years prior to the study. The type of the prescribed drug used as well as the esFigurlished feeding and cleaning protocols in each farm were not changed in the study period. Every farmer was interviewed about management practices such as animal movement and animal treatments using antimicrobials, the construction and functioning of the liquid feeding installation and the routine for its cleaning and disinfection, the ingredients (concentrate, whey or water) of the liquid feed and the use of acidifying feed additives.

Between April and December 2015, feed samples were collected at six time points in the case and at four time points in the control farms. In the case farms, the first sampling was done before treatment, which began within a few days after the pigs weighing 25 to 30 kg entered the fattening unit, in order to know the resistance situation before antimicrobial administration. The remaining five sampling points in time were scheduled on day 6 (i.e. during medication), 12, 18, 36 and 76 after the start of the antimicrobial group treatment. In the control farms, where no short term variation of the antimicrobial resistance situation was to be expected, the second, third and fourth sampling times were fixed on day 8, 14 and 78 after the first sampling. Two samples were collected at each point in time. One sample was collected at the end of the ring line, which is situated right over the mixing tank, when the feed remaining in the ring line between two feeding periods was pumped into the mixing tank at the next feeding. The other sample was collected at the opening of the drop pipe above the feeding trough which was furthest away from the mixing tank. The outflowing liquid feed was collected in a sterile container. As liquid feed in all farms remained in the ring line between feeding times, the feed samples were collected at the morning feeding, thus ensuring to obtain samples of liquid feed that had interacted with the biofilm during the longest time period between two feeding times (11–16 h).

### Microbiological analyses

All feed samples were kept cool during transport and were processed immediately upon arrival in the laboratory. Their pH value was determined using a pH meter (Orion 525, Hügli, Abtwil), and their mould and yeast count was determined according to ISO 21527–1:2008. The number of Enterobacteriaceae and of Enterobacteriaceae which were resistant to sulphonamide and to trimethoprim was determined by means of two serial dilutions with a detection limit of 10 colony forming units/ml (cfu/ml) each. MacConkey agar (Oxoid, Hampshire, UK) and MacConkey agar supplemented with 152 μg/ml sulphamethoxazole (Sigma-Aldrich, St. Louis, USA) and 8 μg/ml trimethoprim (Sigma-Aldrich, St. Louis, USA) were used for the detection of all and of resistant Enterobacteriaceae. The colonies were counted after anaerobic incubation at 37 °C during 24 h. The antimicrobial resistance of the isolated colonies was verified by subculturing one morphologically distinct resistant colony on a MacConkey agar supplemented with 152 μg/ml sulphamethoxazole and 8 μg/ml trimethoprim as described above.

In order to detect resistant Enterobacteriaceae below the detection limit of the quantitative assessment, the two samples collected at the first sampling in every case farm and the two samples collected at the last sampling in every control farm were enriched for Enterobacteriaceae using 10 ml of liquid feed and 90 ml of Enterobacteriaceae Enrichment broth (BD, Franklin Lakes, USA), and the Enterobacteriaceae were subcultured as described above.

### Data processing and statistical analysis

The microbial counts were log10 transformed after adding one to the counts to adjust for zero values in the data. The ratio of resistant/all Enterobacteriaceae was calculated using the untransformed counts. Since most variables did not meet the assumption of normal distribution of residuals even after log transformation, the semiparametric Generalized Estimating Equations (GEE) method was used for the statistical analysis. For multiple comparisons, Tukey contrasts were calculated, using the single step method in order to adjust the *P* values for family-wise error rate. The data were analysed using the open access statistical package R geepack [8].

Three statistical data analyses were made, taking into account all data, the case data only and the control data only. For the statistical evaluation of the combined case and control data, the model contained the fixed factors group affiliation (case, control), sampling time (case: 1, 4, 5, 6; control: 1, 2, 3, 4) and sampling location (ring line, drop pipe), and the random factor farm as well as the interactions group affiliation × sampling location and group affiliation × sampling time. The model for the case data evaluation contained the fixed factors sulphonamide administration (yes, no), chlortetracycline administration (yes, no), sampling time (1, 2, 3, 4, 5, 6), feed acidification (yes, no) and sampling location (ring line, drop pipe), and the random factor farm as well as the interactions sulphonamide × sampling location and chlortetracycline × sampling location. The model for the control case data evaluation contained the fixed factors feed acidification (yes, no), liquid feed component (whey, water), sampling time (1, 2, 3, 4), sampling location (circuit line, drop line) and the random factor farm plus the interaction acidification × liquid feed component.

## Results

### Farm characteristics

All-in, all-out was practised in all case farms and in one control farm, whereas there was a continuous flow of animals in 13 control farms. In seven case farms a sulphonamide-trimethoprim combination was used, whereas a sulphonamide-chlortetracycline-tylosin combination was administered in three farms and chlortetracycline alone was used in three farms. (Table 1). The daily dose per kg body weight recommended by the drug manufacturers was 40 mg sulphonamide, 8 mg trimethoprim and 3.6 mg tylosin. In the mono-drug and in the antimicrobial combination premix the recommended dose of chlortetracycline was 20–30 and 21 mg/kg body weight, respectively. In two farms each the administered dose was one third below and one fourth above the recommended dose respectively. The length of the antimicrobial group therapy varied between 6 and 10 days. The antimicrobial premixes were added to the feed in the mixing tank. The daily dose was administered in five farms at one feeding in the morning, in six farms at two feedings, one in the morning and one in the afternoon or evening, and in two farms at three feedings. In all case farms the amount of liquid feed offered was initially restricted to 50 to 60% of the nutrient requirements and was then gradually increased to 100% of the requirements (corresponding to about 4 l per animal weighing 25 to 30 kg). The gradual increase occurred within 8 to 10 days, in order to ensure the complete drug intake from the first treatment day on. In the farms where sulphonamide and trimethoprim were administered at the recommended dose, the estimated antimicrobial content per ml liquid feed was 500 μg sulphonamide and 100 μg trimethoprim at the beginning and 250 μg sulphonamide and 50 μg trimethoprim towards the end of the treatment period, under the condition that the drug was administered in the whole daily ration.

**Table 1:** Administered drugs, cleaning and feeding protocols in the case and control farms

Farms	drug	Additive for cleaning^a^	Feed acidification^b^	Liquid feed component
Case farms (*n* = 13)	drug1: 7 drug2: 3 drug3: 3	Acid^b^: 4 Soda^c^: 8 No: 1	Yes: 3 No: 10	Whey^d^: 3 Water: 10
Control farms (*n* = 14)	no drug	Acid^b^: 4 Soda^c^: 4 Other: 2 (No cleaning: 4)	Yes: 5 No: 9	Whey^d^: 5 Water: 9

Drug 1: sulphonamide + trimethoprim; drug 2: chlortetracycline + sulphonamide + tylosin; drug 3: chlortetracycline

^a^addition to water for circuit pipeline cleaning or flushing after cleaning

^b^organic acids

^c^caustic soda alone or with sodium hypochlorite

^d^the whey was acidified in two case and in two control farms

The ring lines were cleaned after treatment in four farms and at the end of each fattening period in all case farms. The ring line cleaning interval was about 1 week, 3 months and 6 to 12 months in two, five and three control farms, respectively, while in four control farms the ring lines had not been cleaned for years. In most farms, organic acids or soda were added to the water used for flushing the ring lines (Table 1).

### Evaluation of the case and control farm data

Enterobacteriaceae resistant to sulphonamide and trimethoprim could be isolated without prior enrichment from the feed of all 13 case farms and from the feed of 5 control farms. After enrichment of the 28 samples collected in the control farms, 12 samples of feed flowing from the drop pipe into the feed trough and 8 samples collected at the end of the ring line tested positive for resistant Enterobacteriaceae. In summary, resistant Enterobacteriaceae were detected either without or after enrichment in all farms with the exception of 2 control farms.

In comparison to the control farms, the feed of the case farms contained higher numbers of Enterobacteriaceae (*P* < 0.01), of resistant Enterobacteriaceae (*P* < 0.001) and of moulds (*P* < 0.01), while the yeast count and the pH did not differ between the farms (*P* > 0.05; Table 2). Thirty and 0.02% of the Enterobacteriaceae isolated in the samples of the case and the control farms, respectively, were resistant to sulphonamides and to trimethoprim (*P* < 0.001).

**Table 2:** Enterobacteriaceae (EB), moulds and yeast (log 10 cfu/ml; arithmetic means, standard errors SE in brackets), the proportion of Enterobacteriaceae resistant to sulphonamide and trimethoprim (STrEB), and the pH in the feed. Case vs. control farms and drop pipes vs. ring lines

	Case		Control		*P*	Drop pipes		Ring lines		*P*
EB	2.37	(0.14)	1.37	(0.14)	0.001	2.38	(0.15)	1.53	(0.14)	0.001
STrEB	1.60	(0.14)	0.15	(0.05)	<0.001	1.26	(0.14)	0.74	(0.12)	<0.001
%STrEB	30.0	(3.3)	0.02	(0.01)	<0.001	21.2	(3.3)	17.6	(3.6)	0.93
Moulds	1.55	(0.10)	0.86	(0.11)	0.004	1.56	(0.12)	0.97	(0.10)	0.002
Yeast	5.23	(0.09)	5.81	(0.09)	0.10	5.59	(0.08)	5.36	(0.11)	0.76
pH	5.18	(0.05)	5.03	(0.04)	0.42	5.32	(0.04)	4.93	(0.05)	<0.001

Feed collected from the drop pipes had higher counts of Enterobacteriaceae (*P* < 0.001), of resistant Enterobacteriaceae (*P* < 0.001) and of moulds (*P* < 0.01), and had a higher pH (*P* < 0.001) compared to feed collected at the end of the ring line, whereas the yeast count did not differ (*P* > 0.05) between the two locations (Table 2).

### Case farm data evaluation

**Fig. 1:**
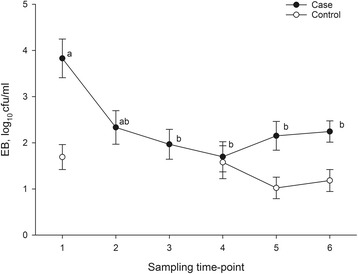
Time course of Enterobacteriaceae (EB) counts in the liquid feed of the case farms and the control farms. Colony forming units (cfu) per ml feed (arithmetic means and standard errors of the samples collected from the drop pipes and the ring lines). Case farms: values within each panel with different superscripts differ (*p* < 0.05)

There was no difference in the Enterobacteriaceae count, the resistant Enterobacteriaceae count and the ratio of resistant to total Enterobacteriaceae in the feed between the 10 farms were sulphonamides were administered and the three farms were chlortetracycline only was used (*P* > 0.05). The total number and the number of resistant Enterobacteriaceae, which showed a similar variation over time (Fig. 1) were high in the first sample collected at both sampling sites, but then decreased and remained rather constant until the last sampling point in time. The ratio of resistant to total Enterobacteriaceae did not differ between the sampling points in time (*P* > 0.05).

Feed acidification was associated with lower counts of total and of resistant Enterobacteriaceae (*P* < 0.001). Feed collected at the opening of the drop pipes was more heavily contaminated with both total and resistant Enerobacteriaceae (*P* < 0.001) than feed collected at the end of the ring line.

### Control farm data evaluation

**Fig. 2:**
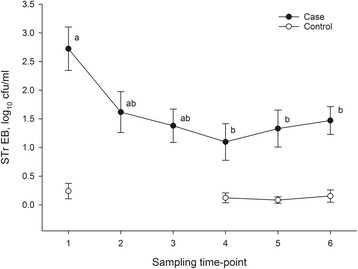
Time course of sulphonamide-trimethoprim resistant Enterobacteriaceae (STr EB) counts in the liquid feed of the case farms and the control farms. Colony forming units (cfu) per ml feed (arithmetic means and standard errors of the samples collected from the drop pipes and the ring lines). Case farms: values within each panel with different superscripts differ (*p* < 0.05)

Neither the liquid component of the diet (whey vs. water) nor the sampling point in time (Fig. 2) influenced the count of total and of resistant Enterobacteriaceae. As in the case farms, feed acidification was associated with a lower Enterobacteriaceae count (*P* < 0.001), and feed collected from the drop pipes contained more Enterobacteriaceae (*P* < 0.001) than the samples collected at the end of the ring line.

## Discussion

In the case farms, which all had an all-in, all-out management with feeding installations temporarily not in use between lots of pigs, the pipelines of the liquid feeding system were cleaned more regularly, and alkalising soda was used more frequently than in the control farms. These management practices are probably the principal reason for the higher Enterobacteriaceae and mould counts in the feed of the case farms. The high initial Enterobacteriaceae counts in the feed samples collected in the case farms in particular, which are close to the acceptable upper limit of 10^4^ cfu/ml [9], may be a consequence of the Enterobacteriaceae proliferation which commonly occurs during the first days of liquid feeding after a thorough cleaning and disinfection of the feeding installation [10]. The disinfection of the pipelines with alkalising products is known to supress the lactobacteria and to favour the growth of Enterobacteriaceae [10, 11]. The aerobic conditions in the drop pipes, which do not contain feed between feeding times, are the most probable cause of the higher Enterobacteriaceae and mould counts and the higher pH in the samples collected above the feed troughs compared to those collected at the end of the ring line.

Because the Enterobacteriaceae count differed between the feed of the case and the control farms for reasons, which are probably not associated with the use of antimicrobials, higher counts of resistant Enterobacteriaceae would be detected in the case farms even in case antimicrobials had not caused a shift in the bacterial population towards resistant Enterobacteriaceae. The differences in the ratio of resistant to total Enterobacteriaceae between the case and the control farms is therefore the relevant criterion by which the effect of the temporary presence of antimicrobials in the pipelines on the prevalence of resistant bacteria in the feed has to be evaluated.

The higher proportion of resistant Enterobacteriaceae in the feed samples of the case farms compared to those from control farms shows that the repeated short-term administration of antimicrobials via the liquid feeding installation is associated with a quantitative shift from a predominantly susceptible towards a sulphonamide and trimethoprim resistant Enterobacteriaceae population. This finding is in accordance with results of studies that link the use of antimicrobials with an increased frequency of resistant bacteria in the environment [12, 13]. However, the difference between the case and the control farms was more pronounced than expected, given the extensive use of sulphonamides and tetracyclines in farm animals over the last 50 years, the frequent occurrence of resistance to these antimicrobials in pathogenic as well as in commensal bacteria [14] and the persistence of the resistance in environmental bacteria [15].

The finding that resistant Enterobacteriaceae could be isolated in the feed of 25 of the 27 farms, although in nine control farms only after enrichment, shows that resistance to sulphonamides plus trimethoprim is widespread in Swiss pig farms. The number of resistant Enterobacteriaceae in the feed would therefore probably rapidly increase if one of the three drugs used in the case farms were administered via the liquid feeding installations of the control farms.

The number of resistant Enterobacteriaceae and the ratio of resistant to total Enterobacteriaceae were not lower in the three case farms where the pigs were treated with the chlortetracycline mono-drug premix compared to the ten farms where sulphonamides were administered. Co-selection, i.e. the selection by chlortetracycline of bacteria which show resistance to sulphonamide and trimethoprim in addition to tetracycline resistance, is the most probable cause for that finding. Wu et al. [16] investigated the prevalence of the sulphonamide resistance genes (*sul1*, *sul2* and *sul3*) in *E. coli* isolated from pig faeces, pig carcasses and human stools and conducted conjugation experiments with a subset of the isolates. They showed that tetracycline resistance genes could be co-transferred with *sul1* and/or *sul2* resistance genes. Gibbons et al. [17] identified the use of antimicrobial combinations containing sulphonamide and trimethoprim as a risk factor for the occurrence of *E. coli* showing resistance to tetracycline in the faeces of pigs. Heller et al. [18] reported that Enterobacteriaceae showing resistance to tetracycline were more prevalent in the feed of farms where sulphonamides and trimethoprim were administered via the liquid feeding installations than in farms where the feeding installations were not used for antimicrobial administration.

The selection process of AMR in a liquid feeding system can potentially occur in antimicrobial containing liquid feed that remains in the circuit pipeline after the feeding or in the biofilm coating the lines of the liquid feeding installation. Whereas bacteria in the liquid feed remaining in the circuit pipeline between feeding cycles are almost completely flushed out during the next feeding, bacteria embedded in the biofilm are very persistant. Because of their high concentration in the feed, in-feed antimicrobials will exert a selective pressure at least on the bacteria near the biofilm-feed interface. Compared to their planktonic counterparts, bacteria enclosed in biofilms profit from a variety of advantages when exposed to antimicrobials, as the extracellular polymeric substances confer, among other things, protection against antimicrobial agents such as antibiotics and disinfectants, and facilitate horizontal gene transfer, i.e. the exchange of mobile genetic elements, which may carry AMR genes, between bacteria [19–23]. Bacteria embedded in a biofilm may be 10 to 1000 times more resistant to antimicrobials than planktonic bacteria [22, 24], suggesting that antimicrobial concentrations may be sub-inhibitory within a biofilm. The use of antimicrobials at sub-inhibitory doses is known to promote the emergence, selection and spread of resistant bacteria [25–27]. The biofilm coating the lines of the liquid feeding system may thus enhance the selection, the persistence and the spread of resistant bacteria, which may be transferred into the liquid feed. The suggested role of the biofilm as a permanent source of resistant bacteria is supported by our finding that the ratio of resistant to total Enterobacteriaceae remained constant during the whole sampling period.

Although the mean number of resistant Enterobacteriaceae at the end of the drop pipes (< 10^2^ cfu/ml) was barely above the detection limit even in the case farms, the fattening pigs, which consumed several litres of liquid feed per day, ingested approximately 10^5^ to 10^6^ resistant Enterobacteriaceae per day. As lactic acid bacteria usually dominate the bacterial flora of liquid feed [11], whose resistance to sulphonamide and trimethoprim was not investigated in this study, the impact of the administration of antimicrobials via the liquid feeding system on the reservoir of sulphonamide and trimethoprim resistance genes and thus the potential of AMR spread among bacteria in the liquid feed was likely to be underestimated. Corpet [28] studied the effect of eating sterilised food on the level of tetracycline resistance among Enterobacteriaceae in human faeces and showed that the ingestion of sterile food leads to a reduction of tetracycline resistance by a factor of 1000. This experiment demonstrated the distinct effect of ingesting commensal food-borne bacteria on the level of AMR in the gut without the oral application of any antimicrobials. This study also suggests that the reduction of the microbial count of animal feed containing a high proportion of resistant bacteria may mitigate the negative effect of such feed on the AMR of the animals’ gastrointestinal bacteria. The addition of organic acids to liquid feed, which have been shown to reduce the number of Enterobacteriaceae and of lactic acid bacteria under experimental conditions [29] and which were associated with reduced Enterobacteriaceae counts in the present study, may help to reduce the number of ingested resistant bacteria in addition to their well-documented beneficial effects on the animals’ intestinal health.

This case-control study has several limitations. Resistance to sulphonamide and trimethoprim was not determined in the individual components of the analysed liquid feed (water, whey, dry feed). It was therefore not possible to assess the proportion of resistant bacteria introduced from outside into the liquid feeding system. The higher cleaning frequency, the more frequent use of alkalising disinfectants and the temporary non-use of the feeding installations in the case farms contributed without doubt to the higher Enterobacteriaceae count in the feed collected in the case farms. Although it would have been preferable to include only farms using the all-in, all-out management system in the study, this was not possible because of the limited number of farms which could be recruited for the study. The authors’ intention was to detect the influence of antimicrobial administration via the liquid feeding installation on the level of resistance to sulphonamide and trimethoprim in average Swiss pig fattening farms. Observational studies including farms with differing management practices have the advantage of being representative of the farming community, allowing the generalisation of the results. On the other hand, it cannot be ruled out that unidentified confounders may have biased the outcome.

## Conclusions

Under the current liquid feeding system management conditions, in-feed antimicrobials which are transported through the lines of liquid feeding installations are selecting resistant bacteria in the biofilm of the lining, which becomes a reservoir of AMR. Resistance to antimicrobials may thus be transmitted via the feed to future batches of fattening pigs even if these are no longer treated with the antimicrobials which have caused AMR in the bacteria colonizing the pipelines. While our findings may not be used to directly link the use of antimicrobials via the liquid feeding system to the emergence of resistant bacteria in the human gut, they should nonetheless, together with the constantly growing body of evidence for the transfer of resistant bacteria from farm animals to humans, and based on the precautionary principle [30], prompt farmers and veterinarians alike to further decrease the amount of antimicrobials used in farm animals. In particular the administration of antimicrobials via liquid feeding systems which are colonised by biofilms have to be avoided unless efficient procedures to reduce the number of resistant bacteria in these linings will be developed.

## Abbreviations

AMR: Antimicrobial resistance
Cfu: Colony forming unit
EB: Enterobacteriaceae
STr: Resistant against sulphonamides and trimethoprim
